# Application of ^99m^Tc-Labeled WL12 Peptides as a Tumor PD-L1-Targeted SPECT Imaging Agent: Kit Formulation, Preclinical Evaluation, and Study on the Influence of Coligands

**DOI:** 10.3390/ph17070906

**Published:** 2024-07-08

**Authors:** Mingxuan Fan, Jingjing Yao, Zuoquan Zhao, Xianzhong Zhang, Jie Lu

**Affiliations:** 1 Key Laboratory of Radiopharmaceuticals, Ministry of Education, College of Chemistry, Beijing Normal University, Beijing 100875, China; 202221150115@mail.bnu.edu.cn (M.F.);; 2 Theranostics and Translational Research Center, Institute of Clinical Medicine, Department of Nuclear Medicine, Peking Union Medical College Hospital, Chinese Academy of Medical Sciences & Peking Union Medical College, Beijing 100730, China

**Keywords:** coligand, Technetium-99m, peptide WL12, PD-L1, SPECT/CT

## Abstract

With the development of PD-1/PD-L1 immune checkpoint inhibitor therapy, the ability to monitor PD-L1 expression in the tumor microenvironment is important for guiding therapy. This study was performed to develop a novel radiotracer with optimal pharmacokinetic properties to reflect PD-L1 expression in vivo via single-photon emission computed tomography (SPECT) imaging. [^99m^Tc]Tc-HYNIC-WL12-tricine/M (M = TPPTS, PDA, ISONIC, 4-PSA) complexes with high radiochemical purity (>97%) and suitable molar activity (from 100.5 GBq/μmol to 300 GBq/μmol) were prepared through a kit preparation process. All ^99m^Tc-labeled HYNIC-WL12 radiotracers displayed good in vitro stability for 4 h. The affinity and specificity of the four radiotracers for PD-L1 were demonstrated both in vitro and in vivo. The results of biodistribution studies displayed that the pharmacokinetics of the ^99m^Tc-HYNIC-conjugated radiotracers were significantly influenced by the coligands of the radiotracers. Among them, [^99m^Tc]Tc-HYNIC-WL12-tricine/ISONIC exhibited the optimal pharmacokinetic properties (*t_1/2α_* = 8.55 min, *t_1/2β_* = 54.05 min), including the fastest clearance in nontarget tissues, highest tumor-to-background contrast (e.g., tumor-to-muscle ratio, tumor-to-blood ratio: 40.42 ± 1.59, 14.72 ± 2.77 at 4 h p.i., respectively), and the lowest estimated radiation absorbed dose, highlighting its potential as a clinical SPECT imaging probe for tumor PD-L1 detection.

## 1. Introduction

Since immunotherapy began in 2011, immune checkpoint inhibitor (ICI) therapy based on the programmed death protein 1 (PD-1)/programmed death protein ligand 1 (PD-L1) signaling pathway has played an increasingly important role in cancer treatment [1]. Recent clinical studies have demonstrated that PD-L1 expression has a significant influence on therapeutic efficacy, and positive responses to PD-1/PD-L1 ICI therapy are only possible in patients with tumors that contain high levels of expressed PD-L1 [2,3]. The main method used in the clinic to assess PD-L1 expression in the tumor microenvironment is invasive biopsy in conjunction with immunohistochemistry (IHC). However, due to the high heterogeneity of PD-L1 expression within both primary tumors and metastases, the ability of IHC detection to accurately evaluate the PD-L1 expression status in real-time and predict treatment response is limited, especially for patients with metastatic diseases [4]. Compared with IHC, nuclear medicine techniques allow real-time, noninvasive visualization of tumor PD-L1 expression in the whole body, which can overcome the shortcomings of IHC methods.

The first-generation PD-L1-targeted radiotracers developed for positron emission tomography (PET) or single photon emission computed tomography (SPECT) imaging were mainly based on anti-PD-L1 antibodies [5,6,7,8,9,10,11,12,13,14,15,16]. For these radiotracers, a longer clearance time is often needed to obtain the optimal contrast of immune imaging. Sometimes, to match the slow clearance rate of antibodies, long half-life nuclides (e.g., ^89^Zr: T_1/2_ = 78.4 h; ^111^In: T_1/2_ = 2.3 d) have to be used to prepare antibody-based radiotracers, which may lead to an increased internal radiation-absorbed dose in patients [17]. Therefore, recent studies have focused mainly on nonantibiotic radiotracers with better pharmacokinetic properties, such as affibodies [18,19], nanobodies [20,21,22], antibody fragments [23], peptides [24,25,26,27,28], and small molecules [29,30,31]. Among these radiotracers, peptide-based PD-L1-targeted radiotracers have attracted increasing interest due to their small size, ease of synthesis and modification, high affinity, and good tumor penetration [32].

The peptide WL12 is a macrocyclic peptide with the sequence cyclo[AcTyr-NMeAla-Asn-Pro-His-Leu-Hyp-Trp-Ser-Trp(Me)-NMeNle-NMeNle-Orn-Cys]-Gly-NH2, which can efficiently inhibit the PD-1/PD-L1 interaction (reported IC_50_ of 22 nM) [33,34]. Recently, several WL12-conjugated derivatives have been radiolabeled with ^64^Cu, ^68^Ga, and ^18^F as positron emission tomography (PET) imaging agents [34,35,36,37,38,39,40,41]. [^64^Cu]WL12 is the first reported WL12-based radiotracer. It displayed PD-L1 specificity both in vitro and in vivo, demonstrating the feasibility of introducing a chelating group into the -*Orn* domain of WL12 [34]. However, high radioactivity accumulation in the kidneys and liver was also observed with [^64^Cu]WL12. Subsequent studies on ^68^Ga- [38] and ^18^F-labeled [36] WL12 peptides were performed to generate PET radiotracers with improved pharmacokinetic properties and image contrast. Among them, ^68^Ga-NOTA-WL12 was evaluated in a first-in-human study [42]. The results demonstrated the feasibility of using ^68^Ga-NOTA-WL12 to detect tumor PD-L1 expression levels in patients with non-small cell lung cancer and its potential to guide ICI therapy. However, ^68^Ga-NOTA-WL12 also displayed high hepatic uptake and a slower clearance rate from the hepatobiliary system, resulting in higher background levels in the abdomen. Compared with PET imaging agents, few WL12-based radiotracers for single-photon emission computed tomography (SPECT) imaging have been published. To date, only Ferro-Flores G et al. reported the preclinical and clinical evaluation of two [^99m^Tc]Tc radiolabeled cyclic peptides ([^99m^Tc]Tc-iPD-L1 and [^99m^Tc]Tc-WL12) in 2023 [43]. Compared to [^99m^Tc]Tc-WL12, [^99m^Tc]Tc-iPD-L1 exhibited greater HCC827 tumor uptake, as well as higher uptake in the liver and kidneys.

Currently, developing a novel WL12-based radiotracer with optimal excretion kinetics is highly desirable to obtain a high target-to-background ratio for PD-L1-positive tumor imaging. In this study, we focused on developing ^99m^Tc-labeled HYNIC-conjugated WL12 for SPECT imaging, which is expected to provide a simple, convenient, and inexpensive diagnostic tool for assessing the status of PD-L1 in cancer patients. ^99m^Tc is the most widely used radionuclide due to its excellent nuclide properties, low cost, and high availability through a ^99^Mo/^99m^Tc generator. Herein, 6-hydrazino nicotinamide (HYNIC) was used as the bifunctional chelating group to conjugate the -*Orn* of WL12 for the following reasons: (1) A high ^99m^Tc-labeling efficiency can be achieved at very low concentrations of HYNIC-conjugated biomolecules [44], which is beneficial for obtaining radiotracers with high molar activity through a kit preparation process while following the requirements of good manufacturing practice (GMP). (2) During the ^99m^Tc-HYNIC radiolabeling process, coligands are essential because they occupy the remaining sites of the ^99m^Tc coordination sphere to form ^99m^Tc-HYNIC complexes with good stability. Due to the significant effect of coligands on their physicochemical properties, such as hydrophilicity, charge, and stability [44,45,46,47,48], a strategy is convenient for optimizing the pharmacokinetic properties of ^99m^Tc-HYNIC complexes via the selection of coligands. In this study, lyophilized kits containing different coligands, tricine/M (M = triphenylphosphine-3,3′,3″-trisulfonic acid trisodium salt (TPPTS), isonicotinic acid (ISONIC), 3,5-pyridine dicarboxylic acid (PDA), or 4-pyridinesulfonic acid (4-PSA), respectively), were developed to prepare ^99m^Tc-WL12 complexes with high radiochemical purity and suitable molar activity. In vitro and in vivo evaluations of these radiotracers were performed, and the results were compared to develop a favorable PD-L1-targeted tumor imaging agent with optimal pharmacokinetic properties for SPECT imaging.

## 2. Results

### 2.1. Peptide Synthesis

**Scheme 1:**
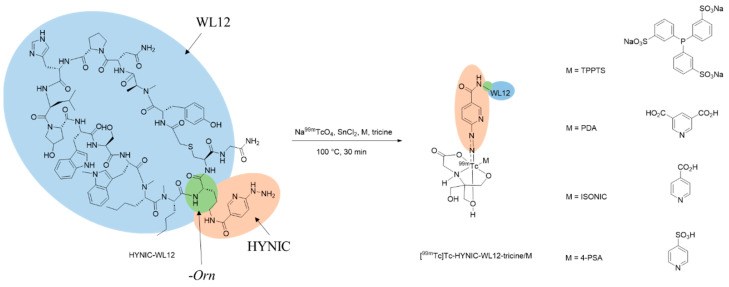
Chemical structure of HYNIC-conjugated WL12 (HYNIC-WL12) and coligands (M = TPPTS, ISONIC, PDA, and 4-PSA), and radiolabeling routes of [^99m^Tc]Tc-HYNIC-WL12-tricine/M complexes.

First, the peptide WL12 was synthesized via solid-phase synthesis. The HYNIC moiety was introduced into the -*Orn* of the WL12 peptide to produce the HYNIC-WL12 peptide (Scheme 1). Both the WL12 and HYNIC-WL12 peptides were purified by high-performance liquid chromatography (HPLC). The identities of the final peptides were confirmed by electrospray ionization mass spectrometry (ESI-MS). The chemical purity of each sample was analyzed by HPLC. The ESI-MS and HPLC results obtained for the final peptides are shown in the Supplemental Information (Figures S1–S4).

### 2.2. Radiochemistry

As shown in Scheme 1, the radiochemical synthesis of [^99m^Tc]Tc-HYNIC-WL12-tricine/M (M = TPPTS or PDA or ISONIC or 4-PSA, respectively) was performed by adding ^99m^Tc eluent into the kit containing the HYNIC-WL12 peptide and coligands and reacting the substances at 100 °C. [^99m^Tc]Tc-HYNIC-WL12-tricine/M (M = TPPTS or PDA or ISONIC or 4-PSA, respectively) was obtained with a high labeling yield (>98%) under the optimized reaction conditions. The molar activity (*A_m_*) of the obtained radiotracer ranged from 1.5 GBq/μmol to 300 GBq/μmol according to the added radioactivity.

As measured by radio-HPLC, the retention times were 9.94 min, 9.77 min, 10.15 min, and 9.71 min for ^99m^Tc-labeled WL12 with tricine/TPPTS, tricine/PDA, tricine/ISONIC, and tricine/4-PSA as coligands, respectively (Table 1). Combined with the results of instant thin-layer chromatography (iTLC), [^99m^Tc]Tc-HYNIC-WL12-tricine/M was obtained with high radiochemical purity (RCP > 97%, Table 1).

The octanol–water partition coefficient (log *D*) of the four radiotracers was determined in a mixture of phosphate-buffered saline (PBS, 0.1 M, pH = 7.4) and *n*-octanol. As shown in Table 1, all four radiotracers were hydrophilic. Among them, [^99m^Tc]Tc-HYNIC-WL12-tricine/TPPTS was the most hydrophilic, with a Log *D* of −1.71 ± 0.09, followed by [^99m^Tc]Tc-HYNIC-WL12-tricine/ISONIC (−0.48 ± 0.04), [^99m^Tc]Tc-HYNIC-WL12-tricine/PDA (−0.39 ± 0.03), and [^99m^Tc]Tc-HYNIC-WL12-tricine/4-PSA (−0.23 ± 0.03).

**Figure 1:**
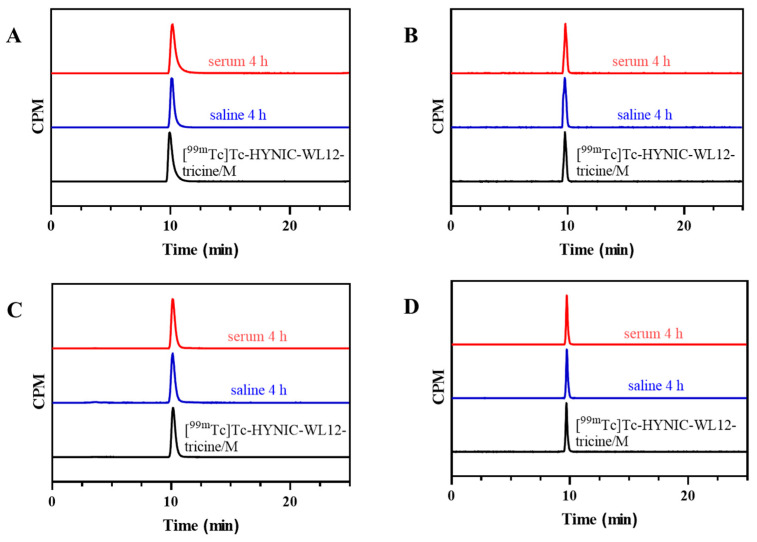
Radio-HPLC profiles of the stability of [^99m^Tc]Tc-HYNIC-WL12-tricine/M incubated in saline and serum for 4 h. ((**A**). M = TPPTS; (**B**). M = PDA; (**C**). M = ISONIC; (**D**). M = 4-PSA).

To evaluate the stability, the radiotracers were incubated in saline at room temperature or mouse serum at 37 °C for 4 h, and the [^99m^Tc]Tc-HYNIC-WL12-tricine/M samples were analyzed by radio-HPLC. As shown in Figure 1, the RCP of all the samples remained above 99%, which demonstrated the good stability of [^99m^Tc]Tc-HYNIC-WL12-tricine/M in vitro.

### 2.3. Cellular Uptake and Blocking Assays

To evaluate the ability of [^99m^Tc]Tc-HYNIC-WL12-tricine/M (M = TPPTS, ISONIC, PDA, and 4-PSA) to target PD-L1, mouse colon cancer MC38 and human PD-L1 gene-transfected MC38 cells (MC38-B7H1) were used. The PD-L1 expression levels in the MC38-B7H1 and MC38 cell lines were evaluated by flow cytometry. As shown in the Supplemental Information (Figure S6), PD-L1 expression was lower in MC38 cells than in MC38-B7H1 cells. Therefore, the affinity and specificity of ^99m^Tc-labeled WL12 radiotracers for PD-L1 were evaluated in the MC38-B7H1 and MC38 cell lines as positive and negative models, respectively.

**Figure 2:**
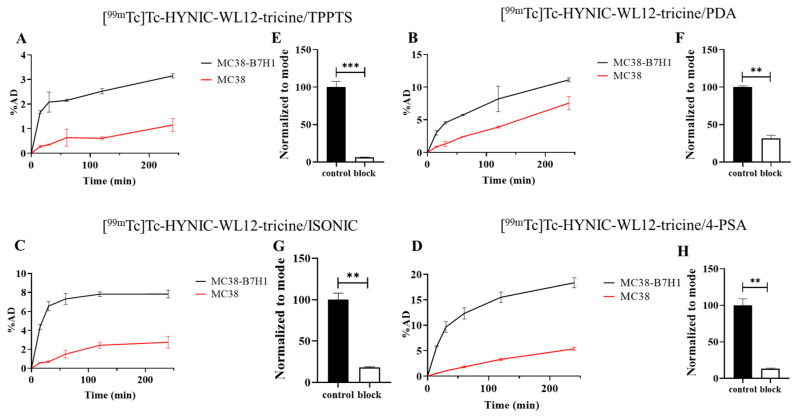
Cellular uptake of [^99m^Tc]Tc-HYNIC-WL12-tricine/M in MC38-B7H1 and MC38 cells (**A**–**D**) was blocked by WL12 in MC38-B7H1 cells (**E**–**H**) (n = 3, ** *p* < 0.01, *** *p* < 0.001).

In cellular uptake studies, the RCP of ^99m^Tc-labeled WL12 radiotracers was greater than 97%, with *A_m_* values ranging from 30 to 60 GBq/μmol. As shown in Figure 2A–D, the cellular uptake of the four ^99m^Tc-HYNIC-WL12 radiotracers in PD-L1-positive MC38-B7H1 cells was significantly greater than that in MC38 cells at each time point, indicating that the uptake of the radiotracers in the cells was dependent on PD-L1 expression.

The PD-L1 specificity of ^99m^Tc-labeled WL12 radiotracers was further confirmed by blocking studies (Figure 2E–H). The uptake of the four radiotracers in MC38-B7H1 cells was clearly blocked by the WL12 peptide (** *p* < 0.01, *** *p* < 0.001). In the presence of 11 μM WL12, the cellular uptake decreased by approximately 94%, 68%, 82%, and 87% for coligands as tricine/M (M = TPPTS or PDA or ISONIC or 4-PSA), respectively.

### 2.4. Ex Vivo and In Vivo Studies

#### 2.4.1. Effect of Molar Activity on Biodistribution

To determine the suitable molar activity of the radiotracer for radiolabeling with a kit formulation, we first investigated the effect of excess ligands on the biological properties of [^99m^Tc]Tc-HYNIC-WL12-tricine/TPPTS using female C57BL/6N mice bearing MC38-B7H1 tumors, which were confirmed to be PD-L1-positive tumor models by immunohistochemistry (Supplementary Information, Figure S7). All animal experiments were approved by the Institutional Animal Care and Use Committee of Beijing Normal University and were carried out in accordance with the Principles of Laboratory Animal Care and the guidelines of the Ethics Committee.

The biodistribution study was performed by the [^99m^Tc]Tc-HYNIC-WL12-tricine/TPPTS injection (RCP > 97%, *A_m_* = 300~1.5 GBq/μmol) obtained via the kit formulation directly or injection further purified by HPLC (to remove excess unlabeled HYNIC-WL12, *A_m_* > 3 TBq/μmol). As shown in Table 2, the uptake of [^99m^Tc]Tc-HYNIC-WL12-tricine/TPPTS in MC38-B7H1 tumors in the “HPLC purification group” was lower than that in the “directly used group (*A_m_* = 300 GBq/μmol)” (6.97 ± 0.50 vs. 16.21 ± 3.12%ID/g) at 2 h post-injection (p.i.). Additionally, the tumor-to-background ratios of [^99m^Tc]Tc-HYNIC-WL12-tricine/TPPTS displayed a similar decreasing trend. Compared with those of the direct injection obtained from the kit (*A_m_* = 300 GBq/μmol), the tumor-to-muscle ratio (T/M) and tumor-to-blood ratio (T/B) of the HPLC-purified injection were reduced from 13.05 ± 3.80 and 5.57 ± 1.07 to 5.82 ± 0.61 and 1.99 ± 0.19, respectively. The results indicated that a small amount of unlabeled ligands exhibited a positive effect on tumor uptake and tumor-to-background ratios.

As shown in Table 2, the tumor uptake of [^99m^Tc]Tc-HYNIC-WL12-tricine/TPPTS in MC38-B7H1 tumors was the highest when the *A_m_* was 300 GBq/mol or 100.5 GBq/μmol (16.21 ± 3.12 and 13.93 ± 1.05%ID/g, respectively, *p* = 0.35). Tumor uptake decreased from 13.93 ± 1.05 to 6.80 ± 1.00%ID/g (*p* < 0.01) when the molar activity decreased from 100.5 to 15 GBq/μmol. Interestingly, the radiotracer with 100.5 GBq/μmol molar activity displayed the highest T/M and T/B ratios. However, the differences in these tumor-to-background ratios were not significant. [^99m^Tc]Tc-HYNIC-WL12-tricine/TPPTS exhibited similar T/M ratios (from 13.05 ± 3.80 to 16.29 ± 2.42) and T/B ratios (from 5.05 ± 1.06 to 7.06 ± 0.45) when the molar activity ranged from 1.5 GBq/μmol to 300 GBq/μmol.

#### 2.4.2. Effect of Coligand on Biodistribution

**Figure 3:**
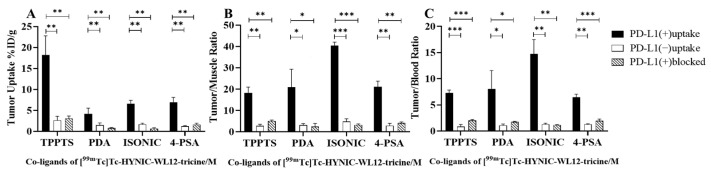
Biodistribution results of [^99m^Tc]Tc-HYNIC-WL12-tricine/M in MC38-B7H1 (PD-L1-positive)- and MC38 (PD-L1-negative)-bearing mice and blocked results in MC38-B7H1-bearing mice at 2 h p.i. (n = 4, * *p* < 0.05, ** *p* < 0.01, *** *p* < 0.001). (**A**) Tumor uptake; (**B**) tumor-to-muscle ratio; and (**C**) tumor-to-blood ratio.

To further confirm the ability of the four radiotracers to target PD-L1 and to investigate the effect of the coligands, biodistribution studies of [^99m^Tc]Tc-radiolabeled HYNIC-WL12 (RCP > 97%; *A_m_*: 100.5–300 GBq/μmol) with different tricine/M coligands (M = TPPTS, PDA, ISONIC, or 4-PSA) were carried out on female C57BL/6N mice bearing MC38-B7H1 or MC38 tumors (confirmed as PD-L1-negative tumors by IHC, Figure S7). As shown in Table 3, Table 4, Table 5 and Table 6, the initial uptake of all four radiotracers was significant, and good retention was achieved in PD-L1-positive MC38-B7H1 tumors. At 0.5 h p.i., the uptake of [^99m^Tc]Tc-HYNIC-WL12-tricine/TPPTS, [^99m^Tc]Tc-HYNIC-WL12-tricine/PDA, [^99m^Tc]Tc-HYNIC-WL12-tricine/ISONIC, and [^99m^Tc]Tc-HYNIC-WL12-tricine/4-PSA in MC38-B7H1 tumors was 11.81 ± 1.53, 5.02 ± 1.61, 7.93 ± 1.19, and 8.22 ± 1.92%ID/g, respectively. At 2 h p.i., more than 80% of the radioactivity accumulation of the radiotracers was still retained in the MC38-B7H1 tumors, which was 18.22 ± 4.57, 4.61 ± 1.32, 6.63 ± 0.80, and 6.96 ± 1.15%ID/g, respectively. Moreover, these radiotracers, [^99m^Tc]Tc-HYNIC-WL12-tricine/M (M = TPPTS or PDA or ISONIC or 4-PSA, respectively), displayed much lower uptake in PD-L1-negative MC38 tumors, with 2.63 ± 0.98, 1.48 ± 0.55, 1.70 ± 0.27, and 1.22 ± 0.19%ID/g at 2 h p.i., respectively (*p* < 0.01, Figure 3). Coinjection of these radiotracers with 50 μg of cold WL12 significantly blocked uptake in MC38-B7H1 tumors (*p* < 0.01, Figure 3), which further confirmed the PD-L1 specificity of [^99m^Tc]Tc-HYNIC-WL12-tricine/M in vivo.

In addition to the uptake in MC38-B7H1 tumors, a high radioactivity accumulation of four radiotracers was found in the liver and kidneys, indicating that the radiotracers may be eliminated from the urinary and hepatic systems. Among them, only [^99m^Tc]Tc-HYNIC-WL12-tricine/TPPTS displayed increased uptake in the kidneys, indicating that the radiotracer was predominantly cleared through the renal pathway. The liver uptake of [^99m^Tc]Tc-HYNIC-WL12-tricine/M (M = TPPTS or PDA or ISONIC or 4-PSA, respectively) was 32.09 ± 1.50, 10.84 ± 1.32, 12.18 ± 1.57, and 32.75 ± 2.29%ID/g at 0.5 h p.i., respectively. At 4 h p.i., the liver uptake decreased by 59.0%, 35.7%, 85.1%, and 38.6%, respectively. Among them, [^99m^Tc]Tc-HYNIC-WL12-tricine/ISONIC exhibited the fastest liver clearance rate. The comparison of main data (e.g., Log *D*, MC38-B7H1 tumor uptake, liver uptake, and kidney uptake) of [^99m^Tc]Tc-HYNIC-WL12-tricine/M (M = TPPTS, PDA, ISONIC, and 4-PSA) was summarized in Table 7.

#### 2.4.3. Dosimetry Estimation

Based on the biodistribution results, time–activity curve fitting and subsequent dose calculation were performed using OLINDA/EXM, version 1.1. The nonlinear curve fitting parameters were applied to derive the best curve fit for the residence time of activity in the source organ. The derived organ residence times were entered in the assumed human model data to derive the absorbed doses to all the organs and the whole-body effective dose, which were generated in mSv/MBq. As shown in Table 8, the effective doses of [^99m^Tc]Tc-HYNIC-WL12-tricine/M (M = TPPTS, PDA, ISONIC, or 4-PSA) were 2.90 × 10^−3^, 2.12 × 10^−3^, 2.24 × 10^−3^, and 2.53 × 10^−3^ mSv/MBq, respectively. The high effective dose of [^99m^Tc]Tc-HYNIC-WL12-tricine/TPPTS was attributed to the high absorbed radiation dose in the kidneys. Among them, [^99m^Tc]Tc-HYNIC-WL12-tricine/ISONIC displayed the lowest organ doses for the liver and kidneys and the lowest effective dose for the whole body.

#### 2.4.4. Pharmacokinetic and Metabolic Analysis

To investigate the blood clearance of the ^99m^Tc-labeled WL12 radiotracers, the pharmacokinetic parameters of [^99m^Tc]Tc-HYNIC-WL12-tricine/M (M = TPPTS and ISONIC) were determined in normal C57BL/6N mice. The pharmacokinetics of [^99m^Tc]Tc-HYNIC-WL12-tricine/M (M = TPPTS, ISONIC) were confirmed as two-compartment models (Supplementary Information, Figure S8). As shown in Table 9, the distribution-phase half-life (*t_1/2_*_α_) values of [^99m^Tc]Tc-HYNIC-WL12-tricine/TPPTS and [^99m^Tc]Tc-HYNIC-WL12-tricine/ISONIC were 59.39 min and 8.55 min, respectively. The clear-phase half-lives (*t_1/2β_*) of [^99m^Tc]Tc-HYNIC-WL12-tricine/TPPTS and [^99m^Tc]Tc-HYNIC-WL12-tricine/ISONIC were 69.32 min and 54.05 min, respectively. The results demonstrated that the blood clearance of [^99m^Tc]Tc-HYNIC-WL12-tricine/ISONIC was faster than that of [^99m^Tc]Tc-HYNIC-WL12-tricine/TPPTS.

**Figure 4:**
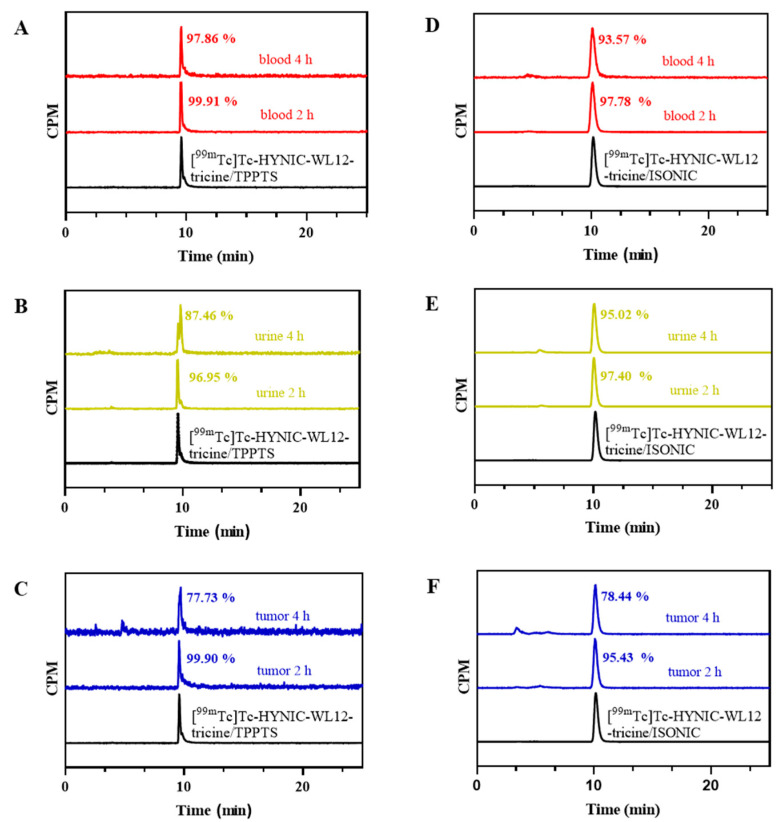
Metabolic stability of [^99m^Tc]Tc-HYNIC-WL12-tricine/TPPTS (**A**–**C**) and [^99m^Tc]Tc-HYNIC-WL12-tricine/ISONIC (**D**–**F**) in tumor-bearing C57BL/6N mice at 2 h and 4 h p.i.

Due to the good in vitro stability of [^99m^Tc]Tc-HYNIC-WL12-tricine/M, we wanted to determine whether the radiotracers remained intact in vivo. Thus, tumor, blood, and urine samples from female MC38-B7H1 tumor-bearing C57BL/6N mice were collected to investigate the metabolic properties of [^99m^Tc]Tc-HYNIC-WL12-tricine/M (M = TPPTS, ISONIC) at 2 h and 4 h p.i. As shown in Figure 4, [^99m^Tc]Tc-HYNIC-WL12-tricine/TPPTS was retained intact in the tumor, blood, and urine samples at 2 h p.i. After 4 h p.i., [^99m^Tc]Tc-HYNIC-WL12-tricine/TPPTS in the blood sample remained unchanged, while in the urine and tumor samples, 87.46% and 77.73%, respectively, of [^99m^Tc]Tc-HYNIC-WL12-tricine/TPPTS remained. Most [^99m^Tc]Tc-HYNIC-WL12-tricine/ISONIC was observed undecomposed in blood, urine, or tumor at 2 h p.i. After 4 h p.i., [^99m^Tc]Tc-HYNIC-WL12-tricine/ISONIC remained unchanged in the blood and urine samples, while 78.44% of the intact [^99m^Tc]Tc-HYNIC-WL12-tricine/ISONIC was found in the tumor samples.

#### 2.4.5. Micro-SPECT/CT Imaging

**Figure 5:**
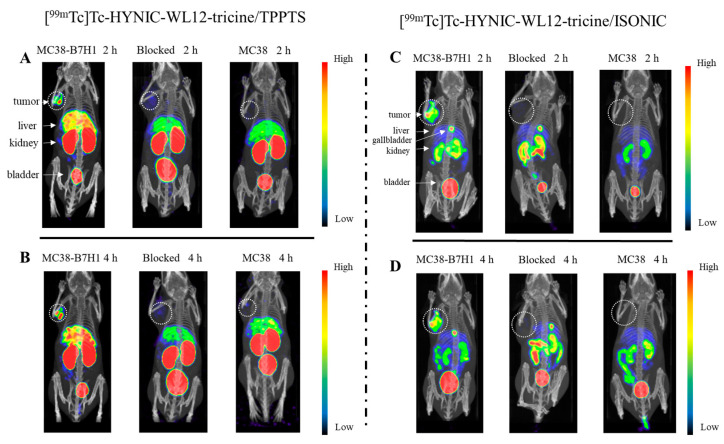
Whole-body micro-SPECT/CT images of tumor-bearing mice injected with [^99m^Tc]Tc-HYNIC-WL12-tricine/TPPTS (**A**,**B**) or [^99m^Tc]Tc-HYNIC-WL12-tricine/ISONIC (**C**,**D**) at 2 h and 4 h p.i. (*A_m_* = 135~150 GBq/μmol).

Micro-SPECT/CT imaging studies of [^99m^Tc]Tc-HYNIC-WL12-tricine/M (M = TPPTS or ISONIC) were performed in mice bearing MC38-B7H1 tumors or MC38 tumors, respectively. The results of micro-SPECT/CT imaging corresponded well with the biodistribution data. With both [^99m^Tc]Tc-HYNIC-WL12-tricine/TPPTS and [^99m^Tc]Tc-HYNIC-WL12-tricine/ISONIC, MC38-B7H1-positive PD-L1-overexpressing tumors were clearly visible at 2 h and 4 h p.i. (Figure 5), while MC38-negative PD-L1-expressing tumors were almost invisible. Blocking with excess cold WL12 peptide resulted in a significant decrease in MC38-B7H1 tumor uptake, confirming the PD-L1 specificity of the radiotracers. Both radiotracers displayed significant radioaccumulation in the bladders of the mice, demonstrating that the two radiotracers were excreted mainly through the urinary system. Compared to [^99m^Tc]Tc-HYNIC-WL12-tricine/TPPTS, [^99m^Tc]Tc-HYNIC-WL12-tricine/ISONIC was significantly accumulated in the gallbladder but exhibited lower uptake in the kidneys and liver at 2 h p.i., indicating that [^99m^Tc]Tc-HYNIC-WL12-tricine/ISONIC is metabolized through a rapid hepatobiliary metabolic pathway.

## 3. Discussion

A simple, efficient, and reproducible kit-based radiolabeling process is essential for the clinical application of ^99m^Tc-radiolabeled radiopharmaceuticals. In this study, a kit formulation was developed for the routine preparation of [^99m^Tc]Tc-HYNIC-WL12-tricine/M (M = TPPTS, PDA, ISONIC, and 4-PSA). During the process of optimizing the kit formulation, a high radiolabeling yield (>97%) of the radiotracers could be obtained with 5 μg of the cold peptide HYNIC-WL12 (the lowest amount tested, Figure S5). However, significant glass surface absorption of these radiotracers was also observed under these low levels of cold peptide. After all the [^99m^Tc]Tc-HYNIC-WL12-tricine/TPPTS solution was removed from the common glass vial, more than 80% of the radioactivity remained on the glass wall. The glass-surface absorption of the radiotracer could be effectively reduced by utilizing silanized glass vials and adding more HYNIC-WL12 peptide as a carrier. However, the addition of cold ligands decreased the molar activity of radiotracers. Generally, high molar radioactivity is needed for receptor-targeted probes due to the limited binding sites and low concentration of biomarkers (usually at the nanomolar level). To evaluate the impact of excessive cold HYNIC-WL12 ligand, a comparative biodistribution experiment was conducted between [^99m^Tc]Tc-HYNIC-WL12-tricine/TPPTS injection with or without excess cold ligand. The results showed that both tumor uptake and tumor-to-background ratios were significantly reduced when excess cold HYNIC-WL12 was removed by further HPLC purification. This result suggested that excessive mass of cold HYNIC-WL12 exerts a positive effect on [^99m^Tc]Tc-HYNIC-WL12-tricine/TPPTS sensitivity in PD-L1-positive tumors. As shown in the biodistribution data of [^99m^Tc]Tc-HYNIC-WL12-tricine/TPPTS with different molar activities (Max: >3 TBq/μmol by HPLC purification, Min: 1.5 GBq/μmol), tumor uptake exhibited a bell-shaped trend with decreasing molar activity. A similar phenomenon was also found for several reported peptide-based radiotracers [49,50,51] and PD-L1-targeted radiolabeled antibodies [52,53]. This was probably because cold HYNIC-WL12 could occupy nonspecific or PD-L1 binding sites in nontarget tissues [8,54], allowing more “free state” radiotracers to accumulate in tumors with high PD-L1 expression. We concluded that a radiotracer with an *A_m_* ranging from 100.5 GBq/μmol to 300 GBq/μmol yielded the best tumor uptake and tumor-to-background contrast. At the typical radiopharmaceutical dose (740–1110 MBq), a kit containing 15 μg of HYNIC-WL12 peptide in a silanized glass vial would be suitable for routine clinical ^99m^Tc radiolabeling.

The results of the IC_50_ determination (as shown in Table S1) displayed that the introduction of the HYNIC moiety in the -*Orn* of WL12 has little influence on the affinity of HYNIC-WL12 for the PD-L1 protein. The results of in vitro cellular assays further demonstrated that four ^99m^Tc-labeled HYNIC-WL12 radiotracers bind to tumor cells in a PD-L1 expression-dependent manner. The cellular uptake of the four radiotracers in MC38-B7H1 cells (PD-L1-positive) was approximately 2.38–6.73-fold higher than that in MC38 cells (PD-L1-negative), which could also be significantly blocked by the addition of the WL12 peptide (*p* < 0.01). The uptake of [^99m^Tc]Tc-HYNIC-WL12-tricine/M (M = TPPTS or PDA or ISONIC or 4-PSA) in MC38-B7H1 tumors was 18.22 ± 4.57, 4.61 ± 1.32, 6.63 ± 0.80, and 6.96 ± 1.15%ID/g at 2 h p.i., respectively, which was 3.11–6.93-fold greater than that in MC38 tumors at the same time points (Figure 3, 2.63 ± 0.98, 1.48 ± 0.55, 1.70 ± 0.27, and 1.22 ± 0.19%ID/g, respectively). The difference in radioactive uptake between the two tumor models was consistent with the IHC staining results, in which PD-L1 expression in the MC38-B7H1 tumors was higher than that in the MC38 tumors (Figure S7). In addition, radioactive accumulation in MC38-B7H1 tumors was reduced by approximately 76.44–89.44% in the blocking group (Figure 3, *p* < 0.01). These results suggested that the tumor uptake of [^99m^Tc]Tc-HYNIC-WL12-tricine/M was PD-L1-specific and associated with the expression level of PD-L1.

**Figure 6:**
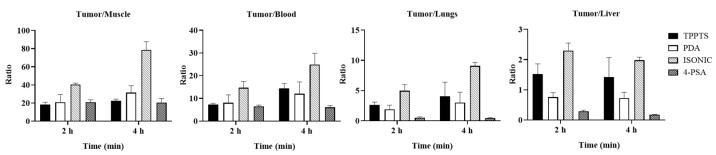
Ratios of tumor/muscle, tumor/blood, tumor/lung, and tumor/liver for [^99m^Tc]Tc-HYNIC-WL12-tricine/M (M = TPPTS, PDA, ISONIC, or 4-PSA, respectively) at 2 h and 4 h p.i.

Despite their high affinity and specificity for PD-L1, the four ^99m^Tc-labeled HYNIC-WL12 radiotracers displayed significantly different pharmacokinetic properties due to their different coligands (Table 3, Table 4, Table 5 and Table 6). Among them, [^99m^Tc]Tc-HYNIC-WL12-tricine/TPPTS exhibited the highest and most increased uptake in MC38-B7H1 tumors (11.81 ± 1.53%ID/g at 0.5 h p.i. and 18.22 ± 4.57%ID/g at 2 h p.i.). The tumor uptake of [^99m^Tc]Tc-HYNIC-WL12-tricine/ISONIC and [^99m^Tc]Tc-HYNIC-WL12-tricine/4-PSA were comparable (7.93 ± 1.19%ID/g vs. 8.22 ± 1.92%ID/g at 0.5 h p.i., respectively), and over 80% of the radioactive accumulation in the MC38-B7H1 tumors still remained at 2 h post-injection. The tumor uptake of [^99m^Tc]Tc-HYNIC-WL12-tricine/PDA was the lowest (5.02 ± 1.61%ID/g at 0.5 h p.i. and 4.61 ± 1.32%ID/g at 2 h p.i.). As shown in Table 7, all four radiotracers displayed the highest initial radioactivity accumulation in the kidneys. However, their kidney clearance rates were significantly different and decreased in the following order: [^99m^Tc]Tc-HYNIC-WL12-tricine/ISONIC > [^99m^Tc]Tc-HYNIC-WL12-tricine/4-PSA > [^99m^Tc]Tc-HYNIC-WL12-tricine/PDA > [^99m^Tc]Tc-HYNIC-WL12-tricine/TPPTS. After 4 h p.i., the kidney uptake of [^99m^Tc]Tc-HYNIC-WL12-tricine/M (M = TPPTS, PDA, ISONIC or 4-PSA) was 154.67 ± 21.76, 31.75 ± 5.16, 5.34 ± 0.73, and 10.80 ± 2.14%ID/g, respectively. The kidney uptake of [^99m^Tc]Tc-HYNIC-WL12-tricine/TPPTS was 28.96-fold higher than that of [^99m^Tc]Tc-HYNIC-WL12-tricine/ISONIC at 4 h p.i. In addition, the liver uptake of [^99m^Tc]Tc-HYNIC-WL12-tricine/M (M = TPPTS, PDA, ISONIC, or 4-PSA) was 12.00 ± 1.11, 6.45 ± 2.70, 2.54 ± 0.70, and 23.44 ± 1.44%ID/g at 2 h p.i., respectively, in the following order of coligand M: 4-PSA > TPPTS > PDA > ISONIC. Compared to [^99m^Tc]Tc-HYNIC-WL12-tricine/TPPTS and [^99m^Tc]Tc-HYNIC-WL12-tricine/4-PSA, in which the coligand (M = TPPTS or 4-PSA) contains sulfonic acid groups, [^99m^Tc]Tc-HYNIC-WL12-tricine/ISONIC and [^99m^Tc]Tc-HYNIC-WL12-tricine/PDA (containing 1–2 carboxyl groups in the coligand ISONIC or PDA) exhibited faster clearance in the liver, as well as other nontarget tissues, such as the lungs, spleen, muscle, and blood. Although its tumor uptake was lower than that of [^99m^Tc]Tc-HYNIC-WL12-tricine/TPPTS, [^99m^Tc]Tc-HYNIC-WL12-tricine/ISONIC exhibited the highest tumor-to-background ratios (Figure 6) due to its faster clearance in nontarget tissues.

The effect of coligands on the pharmacokinetic properties of ^99m^Tc-labeled HYNIC-conjugated biomolecules has been discussed in the literature. One popular explanation is related to the influence of different coligands on the lipophilicity of ^99m^Tc-HYNIC complexes [55,56]. Generally, the increased hydrophilicity of radiotracers often leads to increased urinary excretion. In this study, the log *D* values of [^99m^Tc]Tc-HYNIC-WL12-tricine/M (M = TPPTS, ISONIC, PDA, and 4-PSA) were −1.71 ± 0.09, −0.48 ± 0.04, −0.39 ± 0.03, and −0.23 ± 0.03, respectively. [^99m^Tc]Tc-HYNIC-WL12-tricine/TPPTS, which has the highest hydrophilicity, displayed the highest kidney uptake. However, this hypothesis does not explain why [^99m^Tc]Tc-HYNIC-WL12-tricine/TPPTS and [^99m^Tc]Tc-HYNIC-WL12-tricine/4-PSA exhibit significantly different hydrophilicities (log *D* = −1.71 ± 0.09 vs. −0.23 ± 0.03) but display similar liver uptake (32.09 ± 1.50 and 32.75 ± 2.29%ID/g at 0.5 h p.i., respectively) and slower clearance. An alternative explanation may be related to the effect of the coligands on the stability of the ^99m^Tc-HYNIC complexes [32,57,58]. It was reported that the metabolic stability of [^99m^Tc(HYNIC tetramer)(tricine)(TPPTS)] was much better than that of [^99m^Tc(HYNIC tetramer)(tricine)(ISONIC)] and [^99m^Tc(HYNIC tetramer)(tricine) (PDA)], correlating well with the electron-donating capability of coligands, which decreased as follows: TPPTS > ISONIC > PDA. Compared to [^99m^Tc(HYNIC tetramer)(tricine)(TPPTS)], [^99m^Tc(HYNIC tetramer)(tricine)(PDA)] exhibited lower uptake in most organs of interest due to its poor in vivo stability [57]. In this study, compared to [^99m^Tc]Tc-HYNIC-WL12-tricine/ISONIC, [^99m^Tc]Tc-HYNIC-WL12-tricine/TPPTS had greater tumor uptake and slower clearance rates in nontarget tissues, such as the kidneys, liver, and blood (*t_1/2α_* = 8.55 min vs. 59.39 min, Table 9). However, it does not correlate with the fact that both [^99m^Tc]Tc-HYNIC-WL12-tricine/TPPTS and [^99m^Tc]Tc-HYNIC-WL12-tricine/ISONIC displayed good in vitro stability and similar metabolic stabilities in tumor, blood, and urine (Figure 4). Generally, higher hepatic uptake and longer blood retention often indicate greater protein binding of radiotracers [55,56]. It was reported that the sulfonic acid could act as an “albumin binder” to increase the protein binding of ^64^Cu-labeled PD-L1 small molecules [59]. The coligands TPPTS and 4-PSA both contain a “-SO_3_^−^” group. Therefore, we hypothesized that the higher radioactivity accumulation and retention in nontarget tissues of [^99m^Tc]Tc-HYNIC-WL12-tricine/TPPTS and [^99m^Tc]Tc-HYNIC-WL12-tricine/4-PSA might be related to the “-SO_3_^−^” group of coligands.

For PD-L1-targeted diagnostic radiotracers, an important effort is to reduce radioactive accumulation in nontarget tissues, such as the liver, kidneys, and blood, to (1) obtain a high target-to-background ratio for PD-L1-positive tumor imaging and (2) decrease the radiation risk to the main source organs and the whole body of patients. High kidney and liver uptake are common shortcomings of most reported WL12-based radiotracers. For example, almost all ^64^Cu- and ^68^Ga-labeled WL12 radiotracers significantly accumulated in the kidneys, probably due to the renal clearance pathway of radiolabeled peptides. High hepatic uptake was observed for ^64^Cu-WL12 [39] (24.2 ± 2.5%ID/g at 1 h p.i.) and [^18^F]FPy-WL12 [36] (more than 20%ID/g at 2 h p.i.). As seen in the PET/CT images, ^68^Ga-NOTA-WL12 and ^68^Ga-HBED-CC-WL12 accumulated at significantly high levels in the liver [35,42]. Rapid clearance from the liver was observed only with DOTAGA-conjugated [^68^Ga] WL12 [38], the liver uptake of which was 15.1 ± 7.6 at 1 h p.i. and 2.7 ± 0.2%ID/g at 2 h p.i., respectively. Compared with ^64^Cu-WL12 [34] and [^18^F]FPy-WL12 [36], [^68^Ga] WL12 [38] cleared more quickly from blood and muscle, resulting in greater image contrast. Table 10 compares the biodistribution data between [^68^Ga] WL12 and [^99m^Tc]Tc-HYNIC-WL12-tricine/ISONIC. Similar to [^68^Ga] WL12, [^99m^Tc]Tc-HYNIC-WL12-tricine/ISONIC showed rapid excretion in nontarget tissues, such as the liver, blood, and muscle. After 2 h p.i., the liver uptake, T/B ratio, and T/M ratio of [^99m^Tc]Tc-HYNIC-WL12-tricine/ISONIC were comparable to those of [^68^Ga] WL12 (liver uptake: 2.54 ± 0.70%ID/g vs. 2.7 ± 0.2%ID/g; T/B: 14.72 ± 2.77 vs. 16.02 ± 3.40; T/M: 40.42 ± 1.59 vs. 100.47 ± 61.23). Differences were observed in kidney uptake, with [^99m^Tc]Tc-HYNIC-WL12-tricine/ISONIC exhibiting ~50% less kidney uptake than [^68^Ga] WL12. The kidney uptake of [^99m^Tc]Tc-HYNIC-WL12-tricine/ISONIC was 28.84 ± 4.63 and 11.91 ± 2.68%ID/g at 1 h and 2 h p.i., respectively, and for [^68^Ga] WL12, it was 64.7 ± 12.1 and 27.67 ± 4.09%ID/g, respectively, at the same time points. The faster clearance of [^99m^Tc]Tc-HYNIC-WL12-tricine/ISONIC in the hepatobiliary and renal systems may result in a lower radiation risk for patients. As shown in Table 8, compared to the other [^99m^Tc]Tc-HYNIC-WL12 radiotracers, [^99m^Tc]Tc-HYNIC-WL12-tricine/ISONIC exhibited the lowest organ doses for the liver and kidneys. The effective dose of [^99m^Tc]Tc-HYNIC-WL12-tricine/ISONIC was 2.12 × 10^−3^ mSv/MBq, which was much lower than that of ^68^Ga-NOTA-WL12 [42] (1.85 × 10^−2^ mSv/MBq), a WL12-based PET imaging probe applied for first-in-human evaluation.

Due to faster clearance in nontarget tissues and higher T/B, T/M, and tumor-to-liver ratios, [^99m^Tc]Tc-HYNIC-WL12-tricine/ISONIC exhibited greater contrast between the tumor and background in SPECT/CT images. As shown in Figure 5, the MC38-B7H1 tumor (PD-L1-positive) was clearly visualized at 2 h p.i. After coinjection with cold WL12 peptide, the tumor was almost invisible, which was consistent with the trend observed in the biodistribution results, suggesting that [^99m^Tc]Tc-HYNIC-WL12-tricine/ISONIC is worthy of further preclinical development. However, [^99m^Tc]Tc-HYNIC-WL12-tricine/ISONIC displayed significantly lower MC38-B7H1 tumor uptake at 2 h p.i. than that of [^99m^Tc]Tc-HYNIC-WL12-tricine/TPPTS (6.63 ± 0.80%ID/g vs. 18.22 ± 4.57%ID/g). An improvement in the tumor uptake of [^99m^Tc]Tc-HYNIC-WL12-tricine/ISONIC was warranted. As demonstrated in published literature, the modification of different linkers can improve the tumor uptake of radiotracers to varying degrees [60,61,62]. Additionally, researchers have reported that introducing a PEG3 linker enhances the pharmacokinetic and pharmacodynamic characteristics of Al[^18^F]F-labeled NOTA-PCP1 [63]. In future studies, we will focus on improving the tumor uptake of the ^99m^Tc-radiolabeled HYNIC-WL12 peptide by modifying peptides with different pharmacokinetic (PKM)-modifying linkers.

## 4. Materials and Methods

### 4.1. General Information

^99m^Tc was obtained from a ^99^Mo/^99m^Tc generator (Guangzhou Diqi Trading Co., Ltd., Guangzhou, China) and eluted with saline. All chemical reagents and solvents were purchased from commercial suppliers and were used directly without any further purification. More details are described in the Supplemental Information.

### 4.2. Peptide Synthesis

The peptides WL12 and HYNIC-WL12 were synthesized via solid-phase synthesis and identified by both ESI-MS and HPLC. More details are described in the Supplemental Information.

### 4.3. Preparation of [^99m^Tc]Tc-HYNIC-WL12-Tricine/M

Lyophilized kits were prepared for radiolabeling. Each kit contained a lyophilized mixture of 3 mg of tricine, 2 mg of coligand M (M = TPPTS or PDA or ISONIC or 4-PSA, respectively), 20–30 μg of tin (II), succinate/sodium succinate buffer (0.2 M, pH = 4.6), and HYNIC-WL12 peptide (5–80 μg). The lyophilized powder was sealed in a 10 mL vial and stored at −20 °C. The corresponding [^99m^Tc]Tc-HYNIC-WL12-tricine/M complex was prepared by injecting 1–2 mL of Na^99m^TcO_4_ solution (>37 MBq) into the vial and heating for 30 min at 100 °C.

After the reaction, the solution was cooled to room temperature, and the RCP of the obtained [^99m^Tc]Tc-HYNIC-WL12-tricine/M complex was measured by both radio-high-performance liquid chromatography (radio-HPLC) and instant thin-layer chromatography (iTLC). More details are provided in the Supplemental Information.

### 4.4. Partition Coefficient Study (log D)

The log *D* values of [^99m^Tc]Tc-HYNIC-WL12-tricine/M were determined using phosphate-buffered saline (PBS, 0.1 M, pH = 7.4) and *n*-octanol. More details are provided in the Supplemental Information.

### 4.5. Stability Assay

In the in vitro stability study, the radiotracer [^99m^Tc]Tc-HYNIC-WL12-tricine/M (approximately 0.37 MBq) without HPLC purification was placed at room temperature or incubated in 200 μL of mouse serum at 37 °C for 4 h. The samples at each time point were analyzed by radio-HPLC.

### 4.6. In Vitro Cellular Uptake Assays

Mouse colon cancer MC38 cells and human PD-L1 gene-transfected MC38 cells (MC38-B7H1) were used for in vitro cellular uptake studies. The PD-L1 expression levels in the two kinds of cells were evaluated by flow cytometry. The uptake of [^99m^Tc]Tc-HYNIC-WL12-tricine/M by the MC38 and MC38-B7H1 cell lines was studied. Blocking studies were carried out in MC38-B7H1 cells with positive PD-L1 expression. More details are provided in the Supplemental Information.

### 4.7. Ex Vivo and In Vivo Studies

Ex vivo and in vivo evaluations of [^99m^Tc]Tc-HYNIC-WL12-tricine/M were carried out with female MC38-B7H1 or MC38 tumor-bearing C57BL/6N mice. All animal experiments were approved by the Institutional Animal Care and Use Committee of Beijing Normal University and were carried out in accordance with the Principles of Laboratory Animal Care and the guidelines of the Ethics Committee. The ex vivo part included biodistribution, radiation-absorbed dose analysis, pharmacokinetics, and metabolic studies, and the in vivo part mainly involved micro-SPECT/CT imaging. More details are provided in the Supplemental Information.

### 4.8. Data Analysis

The calculation and analysis of the data were performed using GraphPad Prism 8.0. Quantitative data are shown as the mean ± standard deviation (SD). Statistical analysis was performed using the Student’s *t*-test for unpaired data to determine the significance of differences. Differences at the 95% confidence level (*p* < 0.05) were considered statistically significant.

## 5. Conclusions

In summary, a kit formulation containing HYNIC-WL12 in a silanized glass vial has been proposed to obtain the radiotracer [^99m^Tc]Tc-HYNIC-WL12-tricine/M (M = TPPTS, PDA, ISONIC, 4-PSA) with high radiochemical purity and suitable molar activity. [^99m^Tc]Tc-HYNIC-WL12-tricine/M (M = TPPTS, PDA, ISONIC, 4-PSA) displayed good in vitro stability. All four radiotracers showed high affinity and specificity for PD-L1 both in vitro and in vivo. The coligands of ^99m^Tc-HYNIC-conjugated radiotracers had a significant effect on the pharmacokinetic properties of the radiotracers. Among them, [^99m^Tc]Tc-HYNIC-WL12-tricine/ISONIC exhibited the fastest clearance rate in nontarget organs, the highest tumor-to-background ratios, and the lowest estimated radiation absorbed dose, indicating its potential for further clinical applications.

## Figures and Tables

**Table 1:** Radiochemical data for [^99m^Tc]Tc-HYNIC-WL12-tricine/M (M = TPPTS, PDA, ISONIC, 4-PSA).

Coligands (M)	HPLC Retention Time (min)	RCP (%)	Log *D* (n = 3)
TPPTS	9.94	>98.58	−1.71 ± 0.09
PDA	9.77	>97.36	−0.39 ± 0.03
ISONIC	10.15	>98.50	−0.48 ± 0.04
4-PSA	9.71	>98.29	−0.23 ± 0.03

**Table 2:** Biodistribution data of [^99m^Tc]Tc-HYNIC-WL12-tricine/TPPTS with different molar activities in MC38-B7H1 tumor-bearing mice (%ID/g, mean ± SD, n = 4, at 2 h p.i.).

	Without an Excess of Unlabeled HYNIC-WL12/*A_m_* > 3 TBq/μmol	With an Excess of Unlabeled HYNIC-WL12/*A_m_*			
		300 GBq/μmol	100.5 GBq/μmol	15 GBq/μmol	1.5 GBq/μmol
Tissues					
Heart	3.78 ± 0.67	2.31 ± 0.50	1.74 ± 0.28	1.06 ± 0.20	1.01 ± 0.19
Liver	27.96 ± 4.41	20.44 ± 1.64	11.39 ± 0.45	10.93 ± 2.49	9.64 ± 1.81
Spleen	7.75 ± 1.35	7.00 ± 0.73	4.32 ± 0.94	6.22 ± 1.17	4.35 ± 1.09
Lungs	19.86 ± 1.56	7.72 ± 1.46	6.75 ± 1.77	6.75 ± 3.11	4.26 ± 0.49
Kidney	152.58 ± 19.32	157.43 ± 10.52	132.51 ± 3.63	66.49 ± 13.73	55.22 ± 8.71
Stomach	8.90 ± 3.67	3.43 ± 0.86	2.99 ± 0.56	1.78 ± 0.57	1.62 ± 0.37
Bone	2.80 ± 0.40	1.56 ± 0.27	1.42 ± 0.16	0.78 ± 0.32	0.66 ± 0.15
Muscle	1.21 ± 0.15	1.27 ± 0.16	0.98 ± 0.33	0.53 ± 0.16	0.39 ± 0.05
Tumor	6.97 ± 0.50	16.21 ± 3.12	13.93 ± 1.05	6.80 ± 1.00	6.48 ± 1.59
Small intestine	4.77 ± 2.14	1.87 ± 0.60	1.80 ± 0.94	0.64 ± 0.21	0.64 ± 0.06
Large intestine	3.30 ± 2.05	1.34 ± 0.40	1.27 ± 0.31	0.54 ± 0.06	0.48 ± 0.13
Blood	3.53 ± 0.43	2.93 ± 0.41	2.29 ± 0.47	1.39 ± 0.31	1.26 ± 0.19
Thyroid ^a^	0.11 ± 0.01	0.08 ± 0.02	0.04 ± 0.00	0.03 ± 0.01	0.03 ± 0.01
Ratios					
T/M	5.82 ± 0.61	13.05 ± 3.80	16.82 ± 1.18	13.71 ± 3.76	16.29 ± 2.42
T/B	1.99 ± 0.19	5.57 ± 1.07	7.06 ± 0.45	5.05 ± 1.06	5.15 ± 1.03

^a^: %ID.

**Table 3:** Biodistribution data of [^99m^Tc]Tc-HYNIC-WL12-tricine/TPPTS in MC38-B7H1 tumor-bearing mice (%ID/g, mean ± SD, n = 4).

Tissues	0.5 h	1 h	2 h	4 h
Heart	3.75 ± 0.53	2.79 ± 0.27	1.81 ± 0.23	1.97 ± 0.58
Liver	32.09 ± 1.50	23.61 ± 0.63	12.00 ± 1.11	13.15 ± 3.52
Spleen	14.36 ± 2.69	14.79 ± 2.13	4.61 ± 0.84	7.18 ± 2.94
Lungs	11.04 ± 1.10	9.67 ± 2.67	6.83 ± 1.26	5.75 ± 3.98
Kidney	82.84 ± 12.85	95.79 ± 4.49	144.83 ± 21.48	154.67 ± 21.76
Stomach	5.07 ± 2.38	7.28 ± 4.11	3.33 ± 0.71	2.51 ± 2.27
Bone	2.74 ± 0.15	1.89 ± 0.16	1.42 ± 0.16	1.82 ± 0.69
Muscle	1.75 ± 0.39	1.47 ± 0.38	1.00 ± 0.23	0.76 ± 0.12
Tumor	11.81 ± 1.53	16.61 ± 3.61	18.22 ± 4.57	17.25 ± 2.96
Small intestine	2.24 ± 0.30	2.12 ± 0.16	2.19 ± 0.95	2.32 ± 2.36
Large intestine	1.84 ± 0.41	1.33 ± 0.13	1.17 ± 0.29	3.23 ± 1.93
Blood	5.90 ± 0.56	3.90 ± 0.51	2.47 ± 0.46	1.24 ± 0.40
Thyroid ^a^	0.08 ± 0.01	0.07 ± 0.02	0.07 ± 0.05	0.05 ± 0.03

^a^: %ID.

**Table 4:** Biodistribution data of [^99m^Tc]Tc-HYNIC-WL12-tricine/PDA in MC38-B7H1 tumor-bearing mice (%ID/g, mean ± SD, n = 4).

Tissues	0.5 h	1 h	2 h	4 h
Heart	1.91 ± 0.40	0.92 ± 0.08	0.50 ± 0.05	0.33 ± 0.08
Liver	10.84 ± 1.32	6.00 ± 2.56	6.45 ± 2.70	6.97 ± 1.21
Spleen	5.11 ± 1.75	4.05 ± 2.35	4.98 ± 1.30	4.45 ± 1.47
Lungs	7.60 ± 1.26	4.58 ± 0.53	2.52 ± 0.46	1.47 ± 0.45
Kidney	12.18 ± 1.57	37.04 ± 5.77	36.11 ± 2.78	31.75 ± 5.16
Stomach	2.54 ± 0.52	2.00 ± 0.42	1.92 ± 0.14	1.64 ± 0.96
Bone	1.55 ± 0.29	0.75 ± 0.19	0.54 ± 0.06	0.42 ± 0.07
Muscle	1.01 ± 0.18	0.50 ± 0.05	0.23 ± 0.04	0.13 ± 0.01
Tumor	5.02 ± 1.61	4.02 ± 1.12	4.61 ± 1.32	3.98 ± 1.03
Small intestine	1.27 ± 0.21	1.00 ± 0.05	0.87 ± 0.06	0.78 ± 0.19
Large intestine	1.03 ± 0.19	0.62 ± 0.08	1.12 ± 0.39	1.75 ± 0.26
Blood	2.31 ± 1.62	1.44 ± 0.21	0.61 ± 0.13	0.36 ± 0.10
Thyroid ^a^	0.07 ± 0.01	0.04 ± 0.01	0.03 ± 0.01	0.02 ± 0.01

^a^: %ID.

**Table 5:** Biodistribution data of [^99m^Tc]Tc-HYNIC-WL12-tricine/ISONIC in MC38-B7H1 tumor-bearing mice (%ID/g, mean ± SD, n = 4).

Tissues	0.5 h	1 h	2 h	4 h
Heart	1.57 ± 0.18	1.26 ± 0.49	0.36 ± 0.05	0.13 ± 0.03
Liver	12.18 ± 1.57	8.66 ± 2.90	2.54 ± 0.70	1.82 ± 0.21
Spleen	4.17 ± 0.69	3.40 ± 1.08	1.31 ± 0.50	0.86 ± 0.20
Lungs	6.24 ± 0.76	4.73 ± 1.83	1.27 ± 0.29	0.37 ± 0.08
Kidney	45.10 ± 5.19	28.84 ± 4.63	11.91 ± 2.68	5.34 ± 0.73
Stomach	3.60 ± 0.90	3.88 ± 0.59	1.60 ± 0.32	0.50 ± 0.13
Bone	1.03 ± 0.15	0.78 ± 0.26	0.24 ± 0.09	0.21 ± 0.08
Muscle	0.86 ± 0.06	0.65 ± 0.21	0.17 ± 0.01	0.05 ± 0.01
Tumor	7.93 ± 1.19	9.59 ± 1.95	6.63 ± 0.80	3.71 ± 0.52
Small intestine	1.67 ± 0.18	3.42 ± 1.23	1.90 ± 0.13	0.74 ± 0.31
Large intestine	1.02 ± 0.08	0.94 ± 0.29	1.28 ± 0.53	2.72 ± 0.97
Blood	3.07 ± 0.24	1.93 ± 0.43	0.46 ± 0.06	0.14 ± 0.03
Thyroid ^a^	0.09 ± 0.02	0.06 ± 0.01	0.02 ± 0.01	0.01 ± 0.00

^a^: %ID.

**Table 6:** Biodistribution data of [^99m^Tc]Tc-HYNIC-WL12-tricine/4-PSA in MC38-B7H1 tumor-bearing mice (%ID/g, mean ± SD, n = 4).

Tissues	0.5 h	1 h	2 h	4 h
Heart	2.66 ± 0.58	1.37 ± 0.21	0.88 ± 0.14	0.55 ± 0.05
Liver	32.75 ± 2.29	24.57 ± 2.08	23.44 ± 1.44	20.12 ± 2.58
Spleen	12.16 ± 2.73	13.04 ± 1.54	12.77 ± 4.32	10.63 ± 5.21
Lungs	20.85 ± 2.66	15.09 ± 1.93	14.68 ± 4.73	8.51 ± 1.90
Kidney	48.25 ± 5.69	24.64 ± 4.60	19.59 ± 0.58	10.80 ± 2.14
Stomach	3.37 ± 1.01	2.05 ± 0.50	2.09 ± 1.03	2.18 ± 0.74
Bone	2.35 ± 0.31	0.93 ± 0.41	1.21 ± 0.29	1.21 ± 0.25
Muscle	1.16 ± 0.26	0.44 ± 0.06	0.33 ± 0.02	0.23 ± 0.05
Tumor	8.22 ± 1.92	5.70 ± 1.14	6.96 ± 1.15	4.20 ± 1.58
Small intestine	2.33 ± 0.71	2.17 ± 0.29	2.88 ± 0.77	2.15 ± 0.28
Large intestine	1.33 ± 0.39	0.65 ± 0.10	1.54 ± 0.13	4.44 ± 0.82
Blood	4.55 ± 1.28	1.80 ± 0.32	1.06 ± 0.07	0.59 ± 0.06
Thyroid ^a^	0.09 ± 0.02	0.06 ± 0.01	0.06 ± 0.01	0.08 ± 0.01

^a^: %ID.

**Table 7:** Comparison of main data of [^99m^Tc]Tc-HYNIC-WL12-tricine/M (M = TPPTS, PDA, ISONIC, and 4-PSA, respectively).

Parameter	[^99m^Tc]Tc-HYNIC-WL12-Tricine/M			
	M = TPPTS	M = PDA	M = ISONIC	M = 4-PSA
Log *D*	−1.71 ± 0.09	−0.39 ± 0.03	−0.48 ± 0.04	−0.23 ± 0.03
MC38-B7H1 Tumor uptake				
0.5 h (%ID/g)	11.81 ± 1.53	5.02 ± 1.61	7.93 ± 1.19	8.22 ± 1.92
4 h (%ID/g)	17.25 ± 2.96	3.98 ± 1.03	3.71 ± 0.52	4.20 ± 1.58
Liver uptake				
0.5 h (%ID/g)	32.09 ± 1.50	10.84 ± 1.32	12.18 ± 1.57	32.75 ± 2.29
4 h (%ID/g)	13.15 ± 3.52	6.97 ± 1.21	1.82 ± 0.21	20.12 ± 2.58
Kidney uptake				
0.5 h (%ID/g)	82.84 ± 12.85	12.18 ± 1.57	45.10 ± 5.19	48.25 ± 5.69
4 h (%ID/g)	154.67 ± 21.76	31.75 ± 5.16	5.34 ± 0.73	10.80 ± 2.14

**Table 8:** Estimated radiation absorbed dose of [^99m^Tc]Tc-HYNIC-WL12-tricine/M (female, 56.9 kg) based on C57BL/6N mouse studies.

Organ Doses (mSv/MBq)	[^99m^Tc]Tc-HYNIC-WL12-Tricine/M			
	M = TPPTS	M = ISONIC	M = PDA	M = 4-PSA
Liver	6.04 × 10^−3^	2.36 × 10^−3^	3.04 × 10^−3^	7.51 × 10^−3^
Kidneys	2.57 × 10^−2^	4.53 × 10^−3^	8.46 × 10^−3^	5.66 × 10^−3^
Effective Dose (mSv/MBq)	2.90 × 10^−3^	2.12 × 10^−3^	2.24 × 10^−3^	2.53 × 10^−3^

**Table 9:** Major pharmacokinetic parameters of [^99m^Tc]Tc-HYNIC-WL12-tricine/M (M = TPPTS, ISONIC) after administration to normal female C57BL/6N mice.

Parameter	Unit	Value	
		[^99m^Tc]Tc-HYNIC-WL12-Tricine/TPPTS	[^99m^Tc]Tc-HYNIC-WL12-Tricine/ISONIC
AUC (0–t)	mg/L·min	635.95	450.68
AUC (0–∞)	mg/L·min	721.91	463.49
*t_1/2α_*	min	59.39	8.55
*t_1/2β_*	min	69.32	54.05

**Table 10:** Comparison of the biodistribution data between [^68^Ga] WL12 and [^99m^Tc]Tc-HYNIC-WL12-tricine/ISONIC.

Complex	[^68^Ga] WL12	[^99m^Tc]Tc-HYNIC-WL12-Tricine/ISONIC.
Time p.i.(h)	2	2
tumor (%ID/g)	9.89 ± 1.72	6.63 ± 0.80
liver (%ID/g)	2.7 ± 0.2	2.54 ± 0.70
Liver clearance	82.1% (from 1 to 2 h p.i.)	85.1% (from 0.5 to 2 h p.i.)
kidneys (%ID/g)	27.67 ± 4.09	11.91 ± 2.68
T/M	100.47 ± 61.23	40.42 ± 1.59
T/B	16.02 ± 3.40	14.72 ± 2.77
Animal	NSG mice	C57BL/6N mice
Tumor model	CHO-hPD-L1	MC38-B7H1
references	[38]	Present study

## Data Availability

Data are contained within the article.

## Supplementary Materials

The following supporting information can be downloaded at: https://www.mdpi.com/article/10.3390/ph17070906/s1. Figure S1. HPLC analysis of WL12 (t_R_ = 14.08 min; purity, 95.47%). Figure S2. ESI-MS spectrum of WL12 (*m*/*z*: C_91_H_129_N_22_O_20_S, calculated: [M+H]^+^ 1883.22, [M+2H]^2+^ 942.11. Found: [M+H]^+^ 1883.2, [M+2H]^2+^ 942.6). Figure S3. HPLC analysis of HYNIC-WL12 (t_R_ = 14.29 min, purity 95.84%). Figure S4. ESI-MS of HYNIC-WL12 (*m*/*z*: C_97_H_135_N_25_O_21_S, calculated: [M+2H]^2+^ 1009.68, [M+3H]^3+^ 673.45. Found: [M+2H]^2+^, 1009.62, [M+3H]^3+^ 673.48). Table S1. Results of the IC_50_ tests of WL12 and HYNIC-WL12. Figure S5. Effect of different amounts of HYNIC-WL12 on the labeling yield of [^99m^Tc]Tc-HYNIC-WL12-tricine/TPPTS. Figure S6. Flow cytometry results for the MC38 and MC38-B7H1 cell lines (the vertical axis represents normalization). Figure S7. IHC staining results of MC38-B7H1 and MC38 xenograft tumors (scale bar: 50 μm). Figure S8. Time-activity curve of [^99m^Tc]Tc-HYNIC-WL12-tricine/M (M = TPPTS, ISONIC) in the blood of normal C57BL/6N mice after administration of 0.185 MBq of radiotracers (n = 3).
