# Supplementary material for: Application of 99mTc-Labeled WL12 Peptides as a Tumor PD-L1-Targeted SPECT Imaging Agent: Kit Formulation, Preclinical Evaluation, and Study on the Influence of Coligands
Source: Pharmaceuticals (Basel). 2024 Jul 8;17(7):906. doi: 10.3390/ph17070906 (PMC11279916; doi:10.3390/ph17070906)
Supplement: Supplementary file 1 [file pharmaceuticals-17-00906-s001.zip › pharmaceuticals-3077156-supplementary.pdf]

Application of ^{99m}Tc-Labeled WL12 Peptides as a Tumor PD-L1-targeted SPECT Imaging Agent: Kit Formulation, Preclinical Evaluation and Study on the Influence of Coligands

*Mingxuan Fan, Jingjing Yao, Zuoquan Zhao**, *Xianzhong Zhang, Jie Lu**

Supplementary Information

General Information

High-performance liquid chromatography (HPLC) and radio-HPLC were performed using a SHIMADZU system (CL-20AVP) with a C18 column (250×4.6 mm, 5 μm, Kromasil), which was equipped with an SPD-20AUV detector (λ = 220 nm) and a Bioscan flow count 3200 NaI/PMT γ-radiation scintillation detector. The radioactivity of the iTLC was measured on an HRS-1000 technetium analyzer (Huaruison, Beijing, China). The radioactivity of cellular uptake and biodistribution were assessed with a Wizard 2480 gamma counter (PerkinElmer, Singapore). Several compounds, including PD-1-Eu, PD-L1-biotin and dye-labeled acceptor (BPS Bioscience, US), were combined with a 5430 centrifuge (Eppendorf, Germany) and an EnVision microplate reader (Perkin Elmer, China) to analyse the affinity of WL12 and HYNIC-WL12 for the PD-L1 protein. Antibodies against PD-L1 (rabbit, dilution 1:1000, Servicebio, Wuhan, China), HRP-labeled goat anti-rabbit IgG (dilution 1:200, GB23303, Servicebio, Wuhan, China), haematoxylin staining solution (G1004, Servicebio, Wuhan, China) and DAB staining solution (G1212, Servicebio, Wuhan, China) were used for immunohistochemical staining.

Peptide Synthesis

The peptides WL12 and HYNIC-WL12 were synthesized on Rink Amide-MBHA Resin (ChinaPeptides Co., Ltd., Wuhan, China), in which amino acids or HYNIC were connected via cyclization condensation. The results of ESI-MS and HPLC of the final peptides are shown in Fig. S1-4. For HPLC analysis of WL12, phase A was water containing 0.1% trifluoroacetic acid (TFA), and phase B was acetonitrile containing 0.1% trifluoroacetic acid (TFA). The concentration of phase B gradually changed from 30% to 60% from 0.01–20 min. For the analysis of the peptide HYNIC-WL12, phase A was water containing 0.1% trifluoroacetic acid (TFA), phase B was acetonitrile containing 0.1% trifluoroacetic acid (TFA), and the concentration of phase B was gradually changed from 5% to 95% over 0.01–20 min.

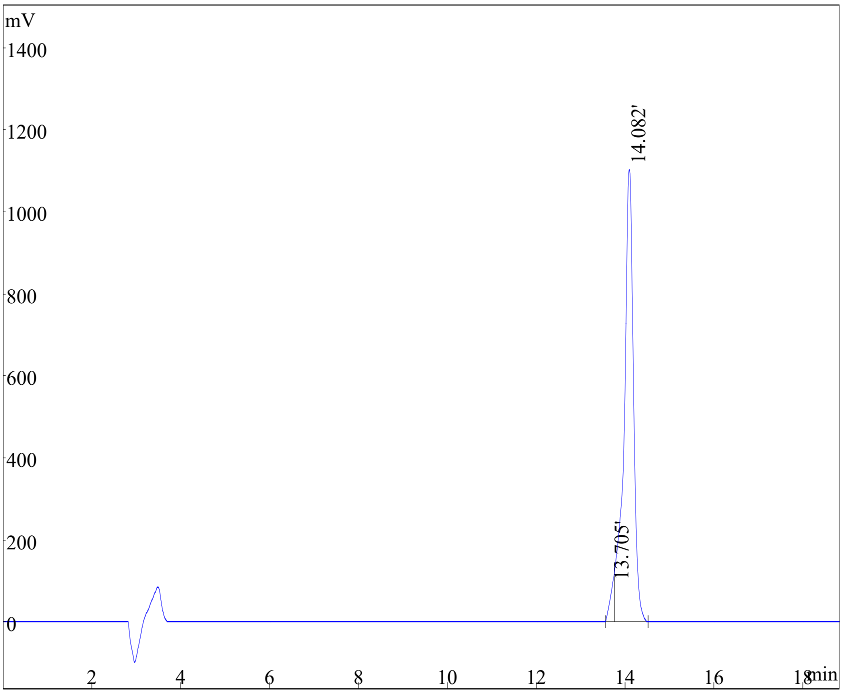

Fig. S1. HPLC analysis of WL12 (t_R = 14.08 min; purity, 95.47%).

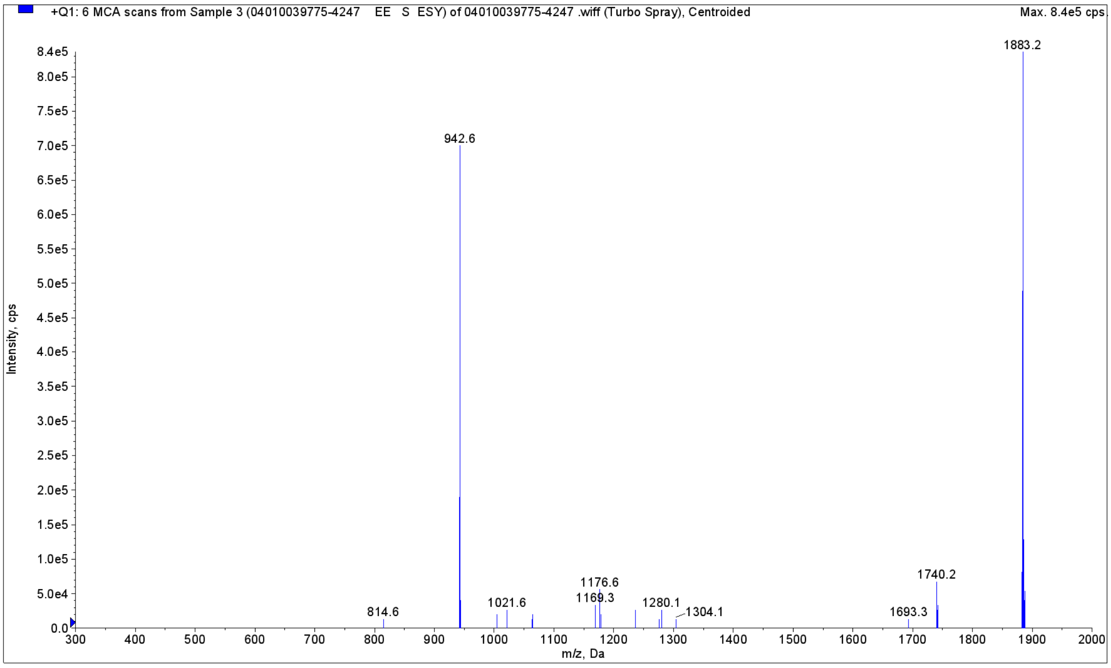

Fig. S2. ESI-MS spectrum of WL12 (m/z: $C_{91}H_{129}N_{22}O_{20}S$, calculated: $[M+H]^+$ 1883.22, $[M+2H]^{2+}$ 942.11. Found: $[M+H]^+$ 1883.2, $[M+2H]^{2+}$ 942.6).

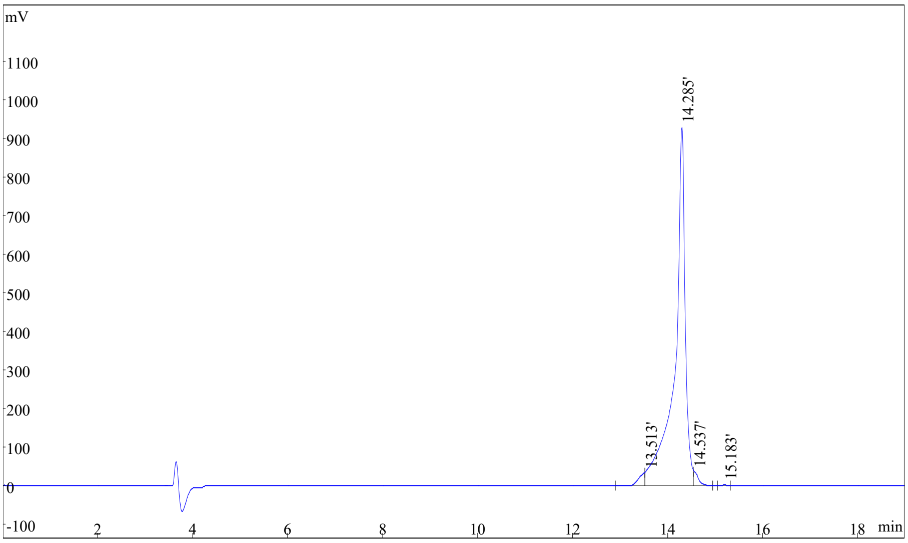

Fig. S3. HPLC analysis of HYNIC-WL12 (t_R = 14.29 min, purity 95.84%).

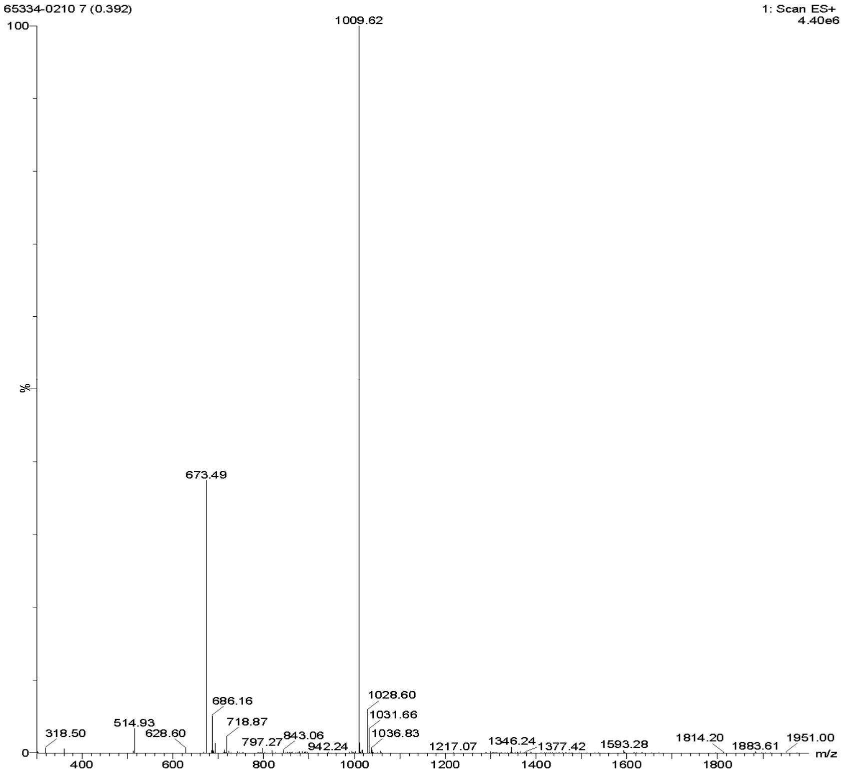

Fig. S4. ESI-MS of HYNIC-WL12 (m/z: $C_{97}H_{135}N_{25}O_{21}S$, calculated: $[M+2H]^{2+}$ 1009.68, $[M+3H]^{3+}$ 673.45. Found: $[M+2H]^{2+}$, 1009.62, $[M+3H]^{3+}$ 673.48).

In Vitro Affinity Assay: Determination of IC_{50}

The affinity of WL12 and HYNIC-WL12 for the PD-L1 protein was determined via a time-resolved fluorescence energy transfer (TR-FRET) assay. In brief, 200 nL of WL12 or HYNIC-WL12 solutions at different concentrations (from 0.5-10000 nM) in 10% ethanol were transferred to 384-well plates on an Echo550

instrument. Then, 5 μL of PD-L1-biotin solution was added, and the plates were centrifuged at 1000 rpm for 30 s and incubated at room temperature. After incubation for 15 min, the mixed solution containing PD-1-Eu and dye-labeled acceptor was added. Then, the plates were centrifuged at 1000 rpm for 30 s and incubated for 90 min at room temperature. Finally, the 665 nm-to-615 nm ratios were read on a microplate reader, and the IC_{50} values of the two peptides were calculated. As shown in Table S1, the peptides WL12 and HYNIC-WL12 displayed similar IC_{50} values (0.15 μM *vs*. 0.17 μM), indicating that introducing HYNIC to WL12 had little effect on the affinity for PD-L1 protein.

Table S1. Results of the IC_{50} tests of WL12 and HYNIC-WL12

	**WL12**	**HYNIC-WL12**
IC_{50} (μM)	0.15	0.17

Radiochemistry

Quality Control of [^{99m}Tc]Tc-HYNIC-WL12-tricine/M

For RP-HPLC analysis of [^{99m}Tc]Tc-HYNIC-WL12-tricine/M, phase A was water containing 0.1% trifluoroacetic acid (TFA), and phase B was acetonitrile containing 0.1% trifluoroacetic acid (TFA). The concentration of phase B was changed as follows: 0.01–2 min, 10%; 2–5 min, from 10% to 90% gradually; 5–18 min, 90%; 18–20 min, from 90% to 10% gradually; and 20–25 min, 10%. Additionally, the radiochemical purity of [^{99m}Tc]Tc-HYNIC-WL12-tricine/M was determined by instant thin layer chromatography on silica gel (iTLC-SG, Agilent, China) using acid-citrate-dextrose solution (ACD solution, pH = 4.5-5.5) as the mobile phase solvent. The R_f values of $^{99m}TcO_4^-$, [^{99m}Tc] colloid and [^{99m}Tc]Tc-HYNIC-WL12-tricine/M were 0.9~1.0, 0.9~1.0 and 0~0.1, respectively.

Effect of HYNIC-WL12 Quantities on the Radiochemical Yield

Taking [^{99m}Tc]Tc-HYNIC-WL12-tricine/TPPTS as an example, the effect of different amounts of HYNIC-WL12 on the radiochemical yield was investigated. The peptide HYNIC-WL12 was formed with a stock

solution (1 mg/mL) in deionized water. To explore the effect of different amounts of HYNIC-WL12, different amounts of WL12-HYNIC stock solutions (5, 10, 20, 40, 50 and 80 μL) were added to vials and radiolabeled with $Na^{99m}TcO_4$ in the presence of 3 mg of tricine, 2 mg of the coligand TPPTS, 20-30 μg of tin (II), and succinate/sodium succinate buffer (0.2 M, pH = 4.6). As shown in Fig. S5, high radiochemical yields were obtained under all reaction conditions.

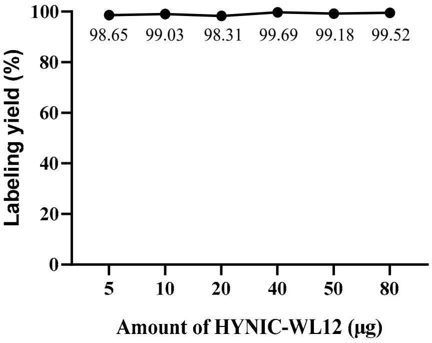

Fig. S5. Effect of different amounts of HYNIC-WL12 on the labeling yield of [^{99m}Tc]Tc-HYNIC-WL12-tricine/TPPTS

Partition Coefficient Study (log D)

The octanol–water partition coefficient was determined by mixing 800 μL of phosphate-buffered saline (PBS, 0.1 M, pH = 7.4) and 50 μL of [^{99m}Tc]Tc-HYNIC-WL12-tricine/M solution (0.37 MBq) with 850 μL of *n*-octanol in triplicate. The mixture was thoroughly vortexed for 2 min and then centrifuged (5000 rpm, 3 min) to ensure that the layers were separated. Finally, each phase (200 μL×3) was measured by a γ-counter. The partition coefficient (Log *D*) was calculated based on the following equation:

Log D = Lg (radioactivity in n-octanol/radioactivity in aqueous layer)

Cell Culture and Animal Models

Mouse colon cancer MC38 and human PD-L1 gene-transfected MC38 cells (MC38-B7H1) were purchased from Cell Resource Center (Beijing, China) and cultured in Roswell Park Memorial Institute (RPMI) 1640 medium supplemented with 10% foetal bovine serum (FBS) and 1% penicillin–streptomycin at 37 °C in a

humidified atmosphere containing 5% CO_2.

Female C57BL/6N mice weighing 18-20 g were purchased from Charles River (China). The unilateral xenografts were implanted with MC38-B7H1 or MC38 cells (1×10^6) in 100 μL of RPMI-1640 in the left axilla of C57BL/6N mice. When the diameter of the tumors reached approximately 10-15 mm, the tumor-bearing mice were used for further experiments. All animal experiments were approved by the Institutional Animal Care and Use Committee of Beijing Normal University and were carried out in accordance with the Principles of Laboratory Animal Care and the guidelines of the Ethics Committee.

Flow Cytometry Analysis of PD-L1 Expression in Cell Lines

The PD-L1 surface expression levels on the two kinds of cells were evaluated by flow cytometry by directly staining 2×10^5 cells in 100 μL of staining buffer (1 mL of FBS in 100 mL of PBS, pH = 7.4) with PE-conjugated mouse anti-human CD274 for 20 min at 4 °C. The cells were then washed and analysed by flow cytometry, with staining buffer serving as a blank control and PE-conjugated mouse IgG1 serving as an isotype control. The data were analysed by FlowJo 7.6.1. As shown in Fig. S6, PD-L1 expression in MC38-B7H1 cells was greater than that in MC38 cells.

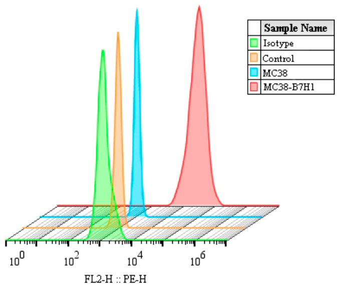

Fig. S6. Flow cytometry results for the MC38 and MC38-B7H1 cell lines (the vertical axis represents normalization).

Cellular Uptake and Block Studies

MC38-B7H1 and MC38 cells were plated on 48-well plates (1×10^5 cells per well) 24 hours prior to the experiment. After the cells were washed once with 1 mL of culture media, the average cell number per well of

each plate was counted. For cellular uptake studies, 0.5 mL of 1640 culture media containing 0.111 MBq of [^{99m}Tc]Tc-HYNIC-WL12-tricine/M was added to each well, and then the plate was incubated at 37 °C. After 15 min, 30 min, 60 min, 120 min and 240 min of incubation, the cell culture supernatants were removed, and the cells were washed twice with ice-cold phosphate buffered saline (0.01 M, pH = 7.4, 0.2% bovine serum albumin (BSA)) and lysed with NaOH (1 M, 0.5 mL) for 5 min at 37 °C. For the blocking group, 0.5 mL of culture media containing ~0.111 MBq of [^{99m}Tc]Tc-HYNIC-WL12-tricine/M and 10 μg of WL12 were added to the wells (n = 3). After incubation for 60 min, the media were removed, and the cells were washed twice with ice-cold phosphate-buffered saline (0.01 mol/L, pH = 7.4, 0.2% bovine serum albumin (BSA)) and lysed with NaOH (1 M, 0.5 mL) for 5 minutes at 37 °C. The lysate and added radioactivity standards were counted with a γ-counter, and the uptake results are shown as the percentage of total added dose (% AD) per 0.1 million cells.

Immunohistochemical Staining

To demonstrate PD-L1 expression in solid tumors, immunohistochemical staining of MC38-B7H1 and MC38 xenograft tumors was performed. Two kinds of solid tumors were removed from female C57BL/6N mice, immersed in neutral paraformaldehyde solution and fixed for 24 h. Paraffin-embedded sections were sequentially dewaxed in environmentally friendly dewaxing solution, anhydrous ethanol, and water and then subjected to microwave heating in EDTA (pH = 9.0) solution for antigen restoration. Then, the sections were placed in a 3% hydrogen peroxide solution, incubated for 25 min at room temperature in the dark and washed three times in PBS (pH = 7.4) for 5 min each, after which 3% BSA was added for 30 min at room temperature. The sections were shaken to remove 3% BSA, PBS (1:1000) containing an antibody against PD-L1 was added dropwise, and the sections were flatly placed in a wet box and incubated at 4 °C overnight. The sections were washed three times in PBS (pH = 7.4) for 5 min each time, and HRP-labeled goat anti-rabbit IgG was added dropwise and incubated at room temperature for 50 min. The sections were washed three times in PBS (pH =

7.4) for 5 min each time, and DAB staining solution was added to stain the sections until they were stopped with water. Haematoxylin was added to stain the cell nuclei for 3 min. The sections were then washed with water, dehydrated, blow-dried, and fixed on slides for observation under a microscope for image acquisition and analysis. As shown in Fig. S7, PD-L1 expression in MC38-B7H1 tumors was greater than that in MC38 tumors.

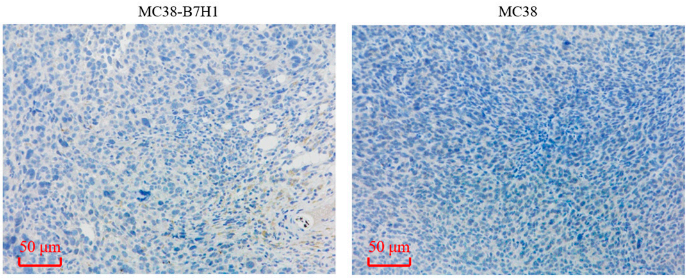

Fig. S7. IHC staining results of MC38-B7H1 and MC38 xenograft tumors (scale bar: 50 μm).

Biodistribution and Radiation-Absorbed Doses Estimates

Effect of molar activity on biodistribution

For biodistribution experiments with different molar activities, MC38-B7H1 tumor-bearing mice (n = 4) were intravenously injected with 100 μL of [^{99m}Tc]Tc-HYNIC-WL12-tricine/TPPTS (0.185 MBq) with or without HPLC purification. HPLC purification was then performed to remove excess HYNIC-WL12. The appropriate fraction was collected, volatile compounds were removed under reduced pressure, and the residue was dissolved in saline for injection. Cold HYNIC-WL12 was determined by comparison with standards containing trace amounts of HYNIC-WL12. The final molar specificity of [^{99m}Tc]Tc-HYNIC-WL12-tricine/TPPTS was calculated as >3 TBq/μmol. For radiotracers without HPLC purification, the molar activity of [^{99m}Tc]Tc-HYNIC-WL12-tricine/TPPTS ranged from 1.5 GBq/μmol to 300 GBq/μmol. The mice were sacrificed after 2 h, and the tumors and related organs of interest were harvested and weighed. Then, the organs were counted by a γ counter, and the radioactivity was calculated and is expressed as the percentage of the injected dose per gram of tissue (%ID/g).

Effect of coligand on biodistribution

For biodistribution experiments with different coligands, MC38-B7H1 tumor-bearing mice (n = 4) were intravenously injected with 100 μL of [^{99m}Tc]Tc-HYNIC-WL12-tricine/M (M = TPPTS or PDA or ISONIC or 4-PSA, 0.185 MBq, A_m: from 100.5 GBq/μmol to 300 GBq/μmol). At 0.5 h, 1 h, 2 h and 4 h post injection, the mice were sacrificed. MC38 tumor-bearing mice (n = 4) were injected with 100 μL of [^{99m}Tc]Tc-HYNIC-WL12-tricine/M (M = TPPTS or PDA or ISONIC or 4-PSA, 0.185 MBq) and were sacrificed at 2 h post injection. For blocking, MC38-B7H1-bearing mice were coinjected with 100 μL of [^{99m}Tc]Tc-HYNIC-WL12-tricine/M (M = TPPTS or PDA or ISONIC or 4-PSA, 0.185 MBq) and 50 μg of WL12 peptide. Then, the mice were sacrificed at 2 h post injection.

Dosimetry Estimation

The biodistribution data of [^{99m}Tc]Tc-HYNIC-WL12-tricine/M (M = TPPTS, PDA, ISONIC and 4-PSA) in MC38-B7H1 tumor-bearing mice at four time points were further used to estimate the radiation absorbed dose via the OLINDA/EXM 1.1 software package.

Pharmacokinetics Study and Metabolic Analysis

To further assess the pharmacokinetics of [^{99m}Tc]Tc-HYNIC-WL12-tricine/TPPTS and [^{99m}Tc]Tc-HYNIC-WL12-tricine/ISONIC, normal C57BL/6N mice were injected intravenously with 100 μL of [^{99m}Tc]Tc-HYNIC-WL12-tricine/M (0.185 MBq, M = TPPTS or ISONIC) via the tail vein. Blood samples were collected and weighed after 2 min, 5 min, 10 min, 30 min, 1 h, 2 h and 4 h, (n = 3). The radioactivity of each sample was measured using a γ-counter. The data were calculated and are expressed as the percentage of injected dose per gram of tissue (%ID/g), and blood uptake-time curves were generated and analysed by DAS 2.0 software. The results are shown in Fig. S8.

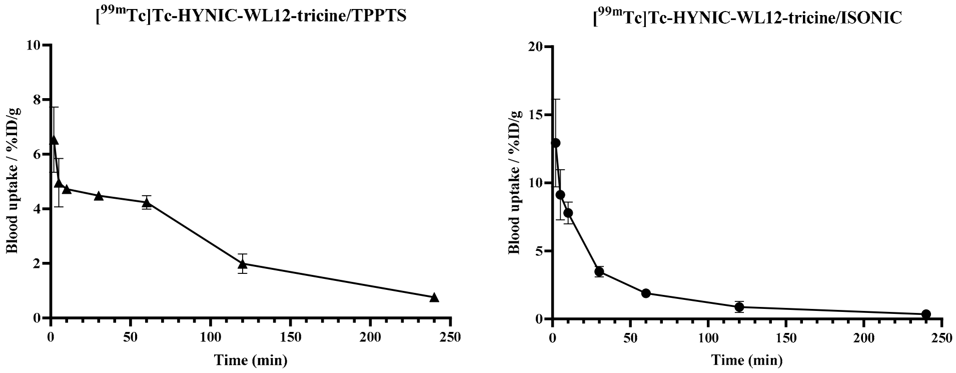

Fig. S8. Time-activity curve of [^{99m}Tc]Tc-HYNIC-WL12-tricine/M (M = TPPTS, ISONIC) in the blood of normal C57BL/6N mice after administration of 0.185 MBq of radiotracers (n = 3).

A metabolic study of [^{99m}Tc]Tc-HYNIC-WL12-tricine/TPPTS and [^{99m}Tc]Tc-HYNIC-WL12-tricine/ISONIC was performed using female C57BL/6N mice bearing MC38-B7H1 tumors. The mice were injected with 200 μL of [^{99m}Tc]Tc-HYNIC-WL12-tricine/M (37 MBq, M = TPPTS or ISONIC) via the tail vein, and blood, urine and tumor samples were collected at 2 h and 4 h, respectively. The blood and urine samples were mixed with acetonitrile to remove the biomacromolecules and then diluted with saline for analysis by radio-HPLC. The MC38-B7H1 tumors were washed with saline and homogenized for 5 min with a LabGEN 7 homogenizer in 2 mL of saline. Protein precipitation was achieved with acetonitrile. The mixture was centrifuged at 12000 rpm for 3 min, and the acetonitrile extracts were collected for radio-HPLC analysis.

Micro-SPECT/CT Imaging

For micro-SPECT/CT imaging, C57BL/6N mice bearing MC38-B7H1 tumors or MC38 tumors were intravenously injected with 200 μL of [^{99m}Tc]Tc-HYNIC-WL12-tricine/M (M = TPPTS or ISONIC, 37 MBq, A_m ranging from 135 GBq/μmol to 150 GBq/μmol). Mice were anaesthetized under 2% isoflurane before scanning and under 1.5% isoflurane during scanning. SPECT/CT imaging was performed at 2 h and 4 h post injection. For blocking, C57BL/6N mice bearing MC38-B7H1 tumors were coinjected with 50 μg of cold WL12 and 200 μL of [^{99m}Tc]Tc-HYNIC-WL12-tricine/M (37 MBq, M = TPPTS or ISONIC) intravenously, and images were also collected at 2 h and 4 h post injection.
